# Genetic diversity of *Streptococcus suis *isolates as determined by comparative genome hybridization

**DOI:** 10.1186/1471-2180-11-161

**Published:** 2011-07-07

**Authors:** Astrid de Greeff, Henk J Wisselink, Freddy M de Bree, Constance Schultsz, Christoph G Baums, Hoa Ngo Thi, Norbert Stockhofe-Zurwieden, Hilde E Smith

**Affiliations:** 1 Infection Biology, Central Veterinary Institute of Wageningen UR (University & Research Centre), Edelhertweg 15, Lelystad, 8219 PH, The Netherlands; 2 Centre for Poverty-Related Communicable Diseases, Academic Medical Centre, Meibergdreef 9, Amsterdam, The Netherlands; 3 Oxford University Clinical Research Unit, Hospital for Tropical Diseases, 190 Ben Ham Tu, Ho Chi Minh City, Vietnam; 4 Institut für Mikrobiologie, Zentrum für Infektionsmedizin, Stiftung Tierärtzliche Hochschule Hannover, Bünteweg 2, Hannover, Germany

## Abstract

**Background:**

*Streptococcus suis *is a zoonotic pathogen that causes infections in young piglets. *S. suis *is a heterogeneous species. Thirty-three different capsular serotypes have been described, that differ in virulence between as well as within serotypes.

**Results:**

In this study, the correlation between gene content, serotype, phenotype and virulence among 55 *S. suis *strains was studied using Comparative Genome Hybridization (CGH). Clustering of CGH data divided *S. suis *isolates into two clusters, A and B. Cluster A isolates could be discriminated from cluster B isolates based on the protein expression of extracellular factor (EF). Cluster A contained serotype 1 and 2 isolates that were correlated with virulence. Cluster B mainly contained serotype 7 and 9 isolates. Genetic similarity was observed between serotype 7 and serotype 2 isolates that do not express muramidase released protein (MRP) and EF (MRP^-^EF^-^), suggesting these isolates originated from a common founder. Profiles of 25 putative virulence-associated genes of *S. suis *were determined among the 55 isolates. Presence of all 25 genes was shown for cluster A isolates, whereas cluster B isolates lacked one or more putative virulence genes. Divergence of *S. suis *isolates was further studied based on the presence of 39 regions of difference. Conservation of genes was evaluated by the definition of a core genome that contained 78% of all ORFs in P1/7.

**Conclusions:**

In conclusion, we show that CGH is a valuable method to study distribution of genes or gene clusters among isolates in detail, yielding information on genetic similarity, and virulence traits of *S. suis *isolates.

## Background

*Streptococcus suis *forms a problem in the swine industry. Clinically healthy sows carry *S. suis *in their nasal cavities and on their tonsils, and transmit the bacteria to their piglets [1], that develop a variety of infections, such as septicaemia, meningitis, polyarthritis, and endocarditis, and often do not survive [2]. *S. suis *occasionally causes meningitis, arthritis or endocarditis in humans. However, recently several large human outbreaks of *S. suis *have been described in China [3,4], and Thailand [5], whilst *S. suis *meningitis has become endemic in Vietnam [6,7], suggesting that isolates that are more virulent to humans have emerged.

The *S. suis *population is very heterogeneous as different serotypes, phenotypes, and genotypes are found. To date 33 capsular serotypes have been described for *S. suis *[2,8] of which serotypes 1, 2, 7, 9, and 14 are most frequently isolated from diseased pigs in Europe [9]. In Northern America, besides these serotypes, serotypes 3 and 8 are frequently isolated from diseased animals [10,11]. On European farms, it was shown that up to 81% of healthy animals carried one or more serotypes simultaneously and different genotypes of the same serotype could be isolated at one timepoint from the same animal [12]. Different phenotypes of serotype 2 were described that differ in their virulence; strains can be differentiated by protein expression of virulence markers muramidase released protein (MRP), extracellular factor (EF) and suilysin (SLY) [13,14]. Besides variation in protein expression observed among *S. suis *strains, large heterogeneity also exists in gene composition [10,15-17]. Recently, the genome sequence of *S. suis *serotype 2 strain P1/7 became available [7] enabling whole genome typing techniques for *S. suis*. In the present study, we performed oligonucleotide-based comparative genome hybridization (CGH) using the genome sequence of strain P1/7 to evaluate gene conservation and diversity among *S. suis *strains. Fifty-five well characterized *S. suis *strains of various serotypes were analyzed in this CGH study. Results from CGH were clustered, and correlated with MLST data, serotyping results, and virulence of strains. We showed that groups of *S. suis *isolates can be identified by their own unique profile of putative virulence genes and regions of difference. Besides, a core genome for *S. suis *was defined.

## Methods

### Bacterial strains and growth conditions

Bacterial isolates are described in Table 1. *S. suis *strains were grown on Columbia agar blood base plates (Oxoid Ltd., London, United Kingdom) containing 6% (vol/vol) horse blood. Cultures were grown in Todd-Hewitt broth (Oxoid). *Escherichia coli *was grown in Luria Broth (Oxoid) and plated on Luria Broth Agar (Oxoid). *S. suis *isolates used in this study were serotyped using the slide-agglutination test [18] before they were used in the study (Table 1). Expression of three virulence markers, MRP, EF, and SLY [19,20] was confirmed for all isolates by Western blot analysis [9] using monoclonal antibodies against MRP, EF [21], or SLY [22] (Table 1).

**Table 1:** Characteristics of bacterial strains used in this study.

Strain	Serotype	Virulence*	ST	MRP^#^	EF^#^	SLY^#^	Clinical source	Reference/source
6388	1	HV	1	S	+	+	Organs	Laboratory collection [32]
6112	1	HV	1	S	+	+	Organs	Laboratory collection [32]
NCTC10273^R1^	1	V	13	-	-	+	Unknown	Laboratory collection [32]
C160	1	ND	1	+	+	+	Brain/septicemia	Laboratory collection [9]
C187	1	ND	132	+	+	+	Tonsil/septicemia	Laboratory collection [9]
OV585	1	ND	13	-	-	+	Unknown	Veterinary Practice Diessen, The Netherlands

12	2	AV	19	-	-	+	Tonsil	Laboratory collection [21]
16	2	AV	19	-	-	+/-	Tonsil	Laboratory collection [21]
25	2	AV	20	-	-	+	Human	Laboratory collection [21]
T15	2	AV	19	-	-	+	Tonsil	Laboratory collection [23]
89-1591	2	ND	25	-	-	-	Unknown	Groupe de Recherché sur les Maladies Infectieuses du Porc, Québec, Canada, [56]
3995	2	ND	1	+	*	-	Unknown	Laboratory collection [16]
3988	2	ND	1	+	*	-	Unknown	Laboratory collection
17	2	WV	8	+	*	+	Tonsil	Laboratory collection
S735^R2^	2	WV	1	+	*	+	Unknown	Laboratory collection CVI
1890	2	ND	133	+	*	+	Unknown	Laboratory collection
3	2	V	1	+	+	+	CNS	Laboratory collection [21]
10	2	V	1	+	+	+	Tonsil	Laboratory collection [21]
22	2	V	1	+	+	+	Human	Laboratory collection [21]
D282	2	V	1	+	+	+	CNS	Laboratory collection [21]
7696	2	ND	1	+	+	+	Joint	Groupe de Recherceh sur les Maladies Infectieuses du Porc, Québec, Canada
P1/7	2	V	1	+	+	+	Unknown	Laboratory collection CVI
BM190	2	ND	1	+	*	+	Human sepsis, meningitis	Hospital for Tropical Diseases, Ho Chi Minh City, Vietnam
BM191	2	ND	1	+	*	+	Human meningitis	Hospital for Tropical Diseases, Ho Chi Minh City, Vietnam
BM334	2	ND	1	-	+	+	Human meningitis	Hospital for Tropical Diseases, Ho Chi Minh City, Vietnam
BM407	2	ND	1	-	*	+	Human meningitis	Hospital for Tropical Diseases, Ho Chi Minh City, Vietnam
FX59	2	ND	1	-	+	+	tonsil	Hospital for Tropical Diseases, Ho Chi Minh City, Vietnam
FX125	2	ND	28	+	-	-	tonsil	Hospital for Tropical Diseases, Ho Chi Minh City, Vietnam
95-8242	2	ND	1	+	+	+	Unknown	Groupe de Recherceh sur les Maladies Infectieuses du Porc, Québec, Canada
89-999	2	ND	25	-	-	-	Unknown	Groupe de Recherceh sur les Maladies Infectieuses du Porc, Québec, Canada, [56]
R75/S2	2	ND	1	+	+	+	Unknown	Groupe de Recherceh sur les Maladies Infectieuses du Porc, Québec, Canada, [56]
98HAH12	2	ND	7	+	+	+	Human TSLS	[41]Hua Dong Research Institute for Medicine of Nanjing Command, Nanjing, China [41]
05ZYH33	2	ND	7	+	+	+	Human TSLS	[41]Hua Dong Research Institute for Medicine of Nanjing Command, Nanjing, China [41]
OV233	2	ND	1	+	+	+	Unknown	Veterinary Practice Diessen, The Netherlands
OV349	2	ND	1	+	+	+	Unknown	Veterinary Practice Diessen, The Netherlands
OV625	2	ND	1	+	+	+	Unknown	Veterinary Practice Diessen, The Netherlands
OV209	4	ND	17	S	-	-	Unknown	Veterinary Practice Diessen, The Netherlands

7711	7	ND	29	-	-	-	CNS	Laboratory collection [9]
7917	7	ND	29	-	-		CNS	Laboratory collection [9]
8039	7	ND	135	-	-	-	CNS	Laboratory collection [9]
C126	7	ND	1	-	-	-	Joint	Laboratory collection [9]
15009	7	ND	89	-	-	-	Unknown	Laboratory collection [9]
A496/98	7	ND	29	-	-	-	Lung, pneumonia	Stiftung Tierärtzliche Hochschule Hannover, Hannover, Germany [25]
Sw123/B2452	7	ND	29	-	-	-	Lung, pneumonia	Stiftung Tierärtzliche Hochschule Hannover, Hannover, Germany [25]
8074^R7^	7	ND	25	-		-	Unknown	Laboratory collection CVI

7997	9	AV	16	*	-	+	Organs	Laboratory collection [9]
8067	9	AV	136	-	-	+	Unknown	Laboratory collection [9]
8017	9	AV	136	-	-	+	CNS	Laboratory collection [9]
7709	9	ND	16	*	-	+	Bacteraemia	Laboratory collection [9]
C132	9	ND	16	*	-	+	Brain/septicemia	Laboratory collection [9]
5973	9	AV	137	*	-	-	CNS	Laboratory collection [9]
22083^R9^	9	ND	82	*	-	-	Unknown	Laboratory collection CVI
7998	9	ND	16	+	-	+	Joint	Laboratory collection [9]
8186	9	ND	138	*	-	-	Tonsil	Laboratory collection [9]

OV640	UT	ND	139	*	-	-	Unknown	Veterinary Practice Diessen, The Netherlands

2840	PA	ND	134	-	*	+	Unknown	Laboratory collection

*Escherichia coli *TG1	NA	NA	NA	NA	NA	NA	NA	Laboratory collection

^Rx ^reference strain of serotype x; UT untypable; PA, poly-agglutination; NA, not applicable; HV, highly virulent; V, virulent; WV, weakly virulent; AV, avirulent; ND, not determined; MRP, muramidase released protein; EF, extracellular factor; SLY, suilysin; +, protein expressed; -, protein not expressed; *, higher MW protein expressed; s, smaller MW protein expressed; +/-, protein weakly expressed; CNS, central nervous system; TSLS, toxic shock-like syndrome

* virulence as determined in an experimental infection in pigs

^# ^phenotype, protein expression

### Experimental infection in pigs

All animal experiments were approved by the ethical committee of the Central Veterinary Institute of Wageningen UR in accordance with the Dutch law on animal experiments.

In this study virulence of *S. suis *isolates was strictly defined by the outcome of experimental infections. To study virulence of *S. suis *serotype 1 and 9 isolates, three successive experiments were performed in pigs. Previous to infection all piglets were tested negative for *S. suis *carriership. In all experiments pigs were allotted to three or four groups each consisting of four or five pigs (Table 2). In the first two experiments seventeen caesarian-derived germfree piglets were housed in stainless steel incubators as described before [21]. Each piglet was infected at the age of 5 days with *Bordetella bronchiseptica *(3 × 10^7 ^CFU, intranasally) to predispose animals for subsequent *S. suis *infection. Two days later animals were infected intranasally with exponentially growing *S. suis *strains (1 × 10^6 ^CFU aerosol).

**Table 2:** Virulence of *Streptococcus suis *serotype 1 and 9 strains determined in pigs.

*S. suis* strain	Sero-type	Dose	No. of pigs	Mortality (%)	Mean time until death (days)	Specific Clinical Signs^1^	Non- Specific Clinical Signs^2^	**Path**. CNS	**Bact**. CNS	**Path**. serosae	**Bact**. serosae	**Path**. joints	**Bact**. joints
6388	1	10^6 ^CFU^$^	5^CD^	100	2	17	37	4	4	4	4	2	4
6112	1	10^6 ^CFU^$^	4^CD^	100	2	7	36	3	4	1	4	0	4
NCTC10273^R1^	1	10^6 ^CFU^$^	4^CD^	100	9.8	6	21	1	2	1	2	4	4
3	2	10^6 ^CFU^$^	4^CD^	50	7	4	67	0	0	4	3	0	0

3	2	10^6 ^CFU^$^	4^CD^	50	7.5	25	87	3	3	3	1	2	3
5973	9	10^6 ^CFU^$^	5^CD^	0	NA	0	0	0	0	2	1	0	0
22083^R9^	9	10^6 ^CFU^$^	4^CD^	0	NA	0	0	0	0	0	0	0	1

22083^R9^	9	10^9 ^CFU	4^SPF^	0	NA	13	0	1	0	0	0	0	1
7997	9	10^9 ^CFU	4^SPF^	25	4	1	2	1	1	0	1	1	2
8067	9	10^9 ^CFU	4^SPF^	0	NA	10	1	0	0	1	0	1	0
28017	9	10^9 ^CFU	4^SPF^	25	1	1	0	1	1	2	0	1	2

^$ ^Animals were predisposed with a *B. bronchiseptica *infection 2 days before infection with *S. suis; *^CD ^caesarean-derived germfree piglets; ^SPF ^specific pathogen free piglets; ^1 ^mean number of observations of nervous signs and lameness in one or more joints/total number of observations × 100%; ^2 ^mean number of observations of inappetence and depression/total number of observations × 100%; Path. Pathology: number of pigs with pathological abnormalities; Bact. Bacteriology: Number of piglets from which *S. suis *is reisolated; NA, animals survived until the end of the experiment; CNS Central nervous system.

In the third experiment, specific pathogen free (SPF) piglets with the age of 6 weeks were infected intranasally with *S. suis *serotype 9 isolates (1 × 10^9 ^CFU) without prior predisposition to *B. bronchiseptica*. Piglets were kept in sternal position and forced to inhale an aerosol produced by an airbrush (Badger, Franklin Park, USA) after anaesthesia with 50% O_2_/50% N_2_O/3% halothane.

In all experiments, piglets were followed clinically with special regard to signs of meningitis and arthritis. Swabs for bacteriological examination were taken daily from the oropharynx and faeces. Pigs were killed either moribund or 18 days post infection at the end of the observation period by intravenous injection of pentobarbiturate followed by exsanguination and necropsy. Tissue specimens from the central nervous system (CNS), serosae, and joints were examined bacteriologically and histologically [21,23].

### Multi Locus Sequence Typing (MLST)

MLST was performed as described by King *et al*. [24]. Alternative primers for *mutS *were used as described previously by Rehm *et al*. [25]. Chromosomal DNA was isolated from stationary growing bacteria as described previously [26]. PCR reactions were performed using Taq PCR Core kit (QIAgen, Hilden, Germany) according to the manufacturer's instructions, using 5 μl of diluted (1:100) chromosomal DNA as template, containing at least 350 ng of DNA. PCR products were visually inspected on 1% agarose gels containing ethidium bromide, and subsequently purified and sequenced by Macrogen (Macrogen, Seoul, Korea). Sequence data were analyzed using Lasergene software (DNAstar, Madison, USA). MLST alleles and resulting STs were assigned using the database on http://ssuis.mlst.net/. New alleles and STs were assigned by the curator of the database. Analysis of ST complexes was performed with eBURST http://www.mlst.net[27].

### *S. suis *oligoarray

A *S. suis *oligoarray (8 × 15 K) containing *in situ *synthesized 60-mers was produced by Agilent Technologies (Santa Clara, USA), according to a custom probe design based on the genome sequence of *S. suis *P1/7 [7]. A total of 7651 unique 60-mers having a theoretical melting temperature of approximately 81°C and representing 1960 ORFs were selected as described by Saulnier *et al*. [28]. Genes were represented by 4 (91%), 3 (4%), 2 (2%), or 1 probe (3%). A total of 25 putative genes were not represented on the array because no unique probe satisfying the selection criteria could be selected.

### Comparative genome hybridization (CGH)

Chromosomal DNA (50 μg) was sheared in 1 ml shearing buffer (TE/10% glycerol), using Nebulizers (Invitrogen, Carlsbad, USA) under 1.7 bar air pressure for 3 minutes to yield fragments between 500 and 1500 bp. DNA was ethanol precipitated, taken up in water and 10 μg of DNA was column purified using Illustra Cyscribe GFX purification kit (GE Healthcare, Uppsala, Sweden) according to instructions of the manufacturer. Differential DNA presence was determined by two-colour fluorescent hybridizations of the corresponding genomic DNAs on the 8 × 15 k *S. suis *oligo array. Genomic DNA of each strain was cohybridized once with the reference strain P1/7, that was always labeled with Cy3. The test strain was consequently labeled with Cy5. Labeling of DNA (2,5 μg) was done using the Bioprime Array CGH Genomic Labeling System (Invitrogen) with slight modifications as described by Molenaar *et al*., 2005 [29]. Labeling efficiency was measured using the Nanodrop (ThermoScientific, Wilmington, USA). Constant amounts of label (25 pmol each) were hybridized to the oligoarray in hybridization buffer of the *In situ *hybridization kit Plus (Agilent Technologies) following instructions of the manufacturer. During hybridization, slides were incubated for 17 h at 65°C under rotation. Slides were washed for 10 min in 6 × SSC/0.05% Triton-X102 at room temperature, followed by 5 min in 0.1 × SSC/0.05% Triton-X102 at 4°C. Slides were dried using pressured air and scanned in a GenePix 4200AL scanner (Molecular Devices, Sunnyvale, USA). Scans were analyzed using GenePix software (Molecular Devices). Local background values were subtracted from the intensity of each spot. Data were normalized using S-Lowess [30] at the webtool accessible from http://bioinformatics.biol.rug.nl/websoftware/s-lowess. Normalized data were imported into Acuity software (Molecular Devices) for further analysis. Cut-off values for presence/absence of genes were empirically determined by comparing microarray results to classic hybridization results using about 100 radioactively labeled probes on spotted chromosomal DNA (data not shown). It was determined that a log ratio above -1.5 indicated the gene was present and very homologous to the gene in P1/7, whereas a log ratio above -4.5 indicated that the gene was present, but variation in nucleotide composition existed among isolates. A ratio between -1.5 and -3 indicated slight variation, whereas a ratio between -3 and -4.5 indicated large variation. A gene was designated "absent" from a genome when all probes for that gene had a normalized log ratio below -4.5.

### Dendrograms

CGH data was clustered using Acuity software to determine similarity of isolates tested in the CGH. Hierarchical clustering of isolates was done by clustering arrays based on ranked correlation coefficients (Spearman's rho), where linkage was determined using the average neighbours method. P-values were calculated by multiscale bootstrap resampling (n = 10000) with the R package pvclust using the average agglomerative method and by the absolute correlative distance measure. The presence of putative virulence genes among isolates, as well as the presence of regions of difference among isolates, was visualized in dendrograms using BioNumerics (Applied Maths, Houston, USA) to study similarity among isolates. These data were analyzed using the Pearson product-moment correlation coefficient. Cluster analysis was done with the unweighted pair group method using arithmetic averages (UPGMA) with a 1% optimization for position tolerance.

### Microarray data

All microarray data have been submitted MIAME complied to ArrayExpress under submission numbers E-MEXP-2531/E-MEXP-2533 http://www.ebi.ac.uk/microarray-as/ae/.

## Results

### Clustering of isolates as determined by CGH

**Figure 1:**
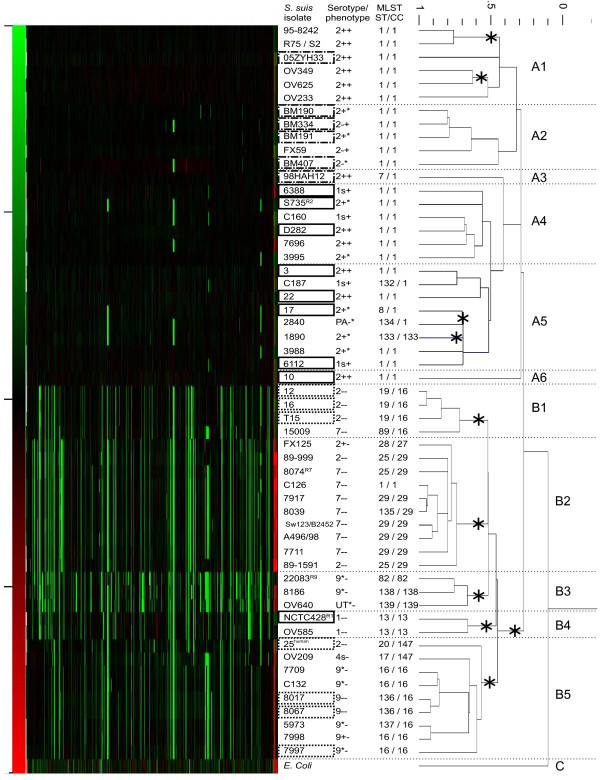
**Dendrogram of normalized CGH results**. *S. suis *strains are listed in the first column, serotype and phenotype (muramidase released protein (MRP) and extracellular factor (EF) expression) in the second column. MLST sequence type (ST) and clonal complex (CC) are listed in the last column. Red color indicates probes that are present in more copies than in P1/7, whereas green color indicates probes that are present in P1/7, and absent in the test strain. Asterisks indicate statistically significant knots. Solid boxed isolates were shown to be virulent or weakly virulent in experimental infections; dotted boxed isolates were shown to be avirulent or very weakly virulent in experimental infections; striped - dotted boxed isolates were isolates from human patients. **^human^**indicates an isolate that was shown to be avirulent in experimental infection, but was isolated from a human patient.

CGH was used to study genomic diversity among *S. suis *isolates. *S. suis *isolates from different serotypes, isolated from different hosts, from different clinical sources, and from different geographical locations were included in the study (Table 1). The dendrogram depicting the CGH data (Figure 1) shows that isolates were divided into 2 clusters, A and B, whereas the negative control *E. coli *strain was assigned to cluster C. This indicates that there are extensive genetic differences between *S. suis *isolates belonging to clusters A and B. Statistical analysis showed that subclustering of isolates in cluster B was highly significant (indicated in Figure 1), whereas subclustering of isolates in cluster A was less significant. This is probably due to high similarity among cluster A isolates. One statistical outlier was identified, isolate 6388 clustered with *E. coli *(p = 0.6) in a separate cluster due to low microarray signals. This was only detected after multiple bootstrap resampling.

Cluster A exclusively contained serotype 1 and 2 isolates, except for one (isolate 2840), indicating that these serotypes are genetically very similar. Isolate 2840 was identified to be poly-agglutinable in a slide agglutination test, although CGH data showed this isolate contains *cps *genes of serotype 2, suggesting the isolate belongs to serotype 2 but does not express (enough) capsule genes sufficiently to be detected in slide agglutination. All isolates in cluster A expressed either EF protein or the larger form EF* protein [16], whereas none of the isolates clustered in group B expressed either of these proteins. MLST analysis showed that with the exception of serotype 2 isolate 1890, all isolates in cluster A belonged to clonal complex 1 (CC1) within which most isolates were found to represent sequence type 1 (ST1) whereas others represented single locus variants of ST1. Six subclusters (A1 - A6) were distinguished in cluster A. Cluster A1 contained MRP^+^EF^+ ^serotype 2 isolates from different geographical locations (Canada, Netherlands and China) that were isolated from humans and pigs, indicating the global spreading of these isolates. Cluster A2 exclusively contained serotype 2 isolates from Vietnam either obtained from human patients or from pigs [6], suggesting these Vietnamese isolates are highly similar to each other. Discrimination of isolates of the subclusters A1 - A6 was based on sequence diversity between genes, rather than on differences in gene content.

In contrast to cluster A, cluster B contained a more divergent, heterogeneous group of isolates. Cluster B contained all serotype 7 and 9 isolates included in this study as well as a number of less virulent serotype 1 and serotype 2 isolates that neither express MRP nor EF. Within cluster B five subclusters were distinguished (B1 - B5). Subclusters B1 and B2 contained all serotype 7 isolates, as well as a number of MRP^-^EF^- ^serotype 2 isolates [21]. The high degree of similarity observed between MRP^-^EF^- ^serotype 2 and serotype 7 isolates could suggest that the MRP^-^EF^- ^serotype 2 isolates originated from serotype 7 isolates by an exchange of capsular genes. This idea is supported by MLST data which showed that most isolates within the clusters B1 and B2 share the same clonal complex (respectively 16 and 29) as well as by AFLP-data in which these isolates also clustered together (data not shown). Cluster B3 was a very heterogeneous group of isolates that seemed to contain isolates that were clustered based on lack of genetic similarity to each other and to other strains. Surprisingly, the reference strain of serotype 9 (22083^R9^) was assigned to cluster B3 as well, at large distance from other serotype 9 isolates in cluster B5. This clearly indicates that the reference strain does not represent the European serotype 9 isolates from the field used in this study. This was confirmed by MLST data, since this reference strain was assigned to ST82, an independent ST, outside a lineage. Cluster B4 contained two serotype 1 isolates among which the reference strain of serotype 1 (NCTC10273^R1^), indicating extensive sequence differences between serotype 1 strains in cluster B4 and serotype 1 strains in cluster A. In contrast to the serotype 1 isolates present in cluster A, both isolates in cluster B4 were negative for expression of MRP and EF and belonged to CC13, whereas all serotype 1 isolates in cluster A belonged to CC1. Therefore, the reference strain for serotype 1 at best represents part of the serotype 1 population. Cluster B5 contained serotype 9 isolates belonging to CC16 as well as a serotype 2 isolate from a human patient and a serotype 4 isolate both belonging to CC147.

### Virulence of *S. suis *isolates of serotype 1 and 9

To be able to study the correlation of gene content of isolates with virulence, we determined the virulence of serotype 1 and 9 isolates used in this study in experimental infections in pigs in comparison to the virulence of serotype 2 strain 3 [21]. The reference strains of serotype 1 and 9 were included in this experimental infection, as well as 2 - 3 field isolates of both serotypes. Table 2 shows that although serotype 1 reference strain NCTC10273^R1 ^showed less clinical signs than serotype 2 strain 3, mortality of serotype 1 reference strain was 100% whereas strain 2 showed only 50% mortality. Four piglets infected with this serotype 1 strain showed pathological abnormalities in joints. Based on morbidity, mortality and pathological abnormalities in > 50% of piglets, isolate NCTC10273^R1 ^is considered virulent, like strain 3. Serotype 1 isolates 6112 and 6388 also showed a mortality rate of 100%. The mean number of days until death of these animals was 2 days, whereas for piglets infected with the serotype 1 reference strain this was 9.8 days. Animals infected with strain 3 showed 50% mortality and a mean number of days until death of more than 7 days post-infection. Isolates 6112 and 6388 induced pathological abnormalities in CNS in 4 out of 5 piglets and 3 out of 5 piglets, respectively. Based on these observations, these serotype 1 isolates are considered more virulent than strain 3 and are therefore considered highly virulent. Serotype 9 isolates did not show any clinical symptoms after an intranasal infection with 10^6 ^CFU (Table 2), whereas strain 3 showed 50% mortality and a mean number of days until death of 7.5. Even an infection dose of 10^9 ^CFU of serotype 9 only induced mild clinical signs, and sparse pathological findings. This led to the conclusion that the serotype 9 isolates tested in our experimental infection model should be considered avirulent, although they can induce mild clinical symptoms at a higher dose.

Virulence of isolates as determined in experimental infections in pigs was depicted in the dendrogram of CGH data (Figure 1). Except for the virulent reference strain of serotype 1 that was assigned to cluster B4, all avirulent isolates were assigned to cluster B, whereas all virulent, highly virulent and weakly virulent isolates were assigned to cluster A. MLST data confirmed these findings, since all isolates in cluster A, except for isolate 1890, belonged to MLST CC1 that was described to have a strong correlation with invasive diseases, like septicemia, meningitis and arthritis [24]. All Asian human isolates that were obtained from meningitis and sepsis patients were assigned to cluster A as well. The only Dutch human isolate from a meningitis patient (isolate 25) was shown to be avirulent in an experimental infection in piglets, and was assigned to cluster B, clearly indicating that this isolate is genetically distinct from the highly virulent Asian human isolates [3,4].

### Distribution of putative virulence related genes among *S. suis *serotype 2 isolates

**Figure 2:**
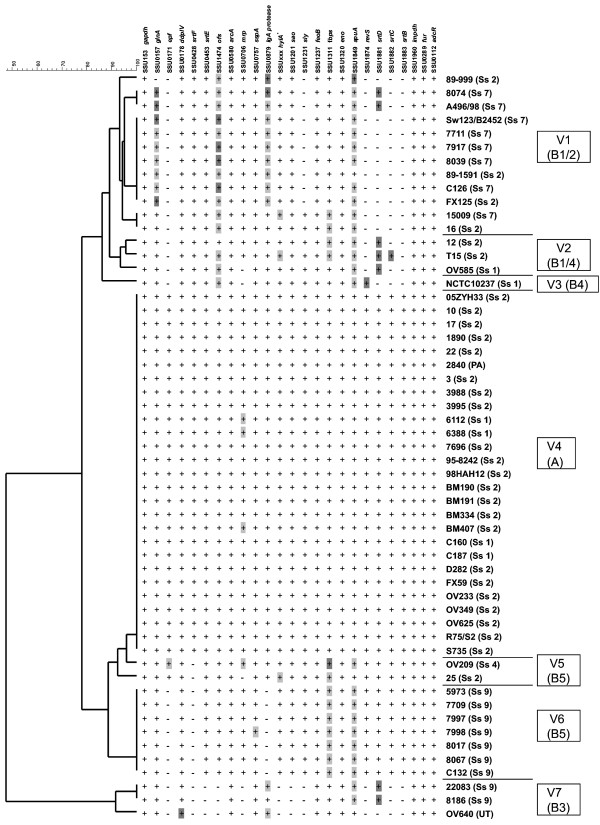
**Presence/absence of 25 putative virulence genes represented in a dendrogram**. Naming (SSU numbering) is derived from the annotated genome sequence of P1/7 [7]. Presence of 25 described putative virulence factors was studied: muramidase released protein (*mrp*), and extracullar factor (*epf*) [13], suilysin (*sly*) [20], sortases (*srtA*, *srtBCD*, *srtF*) [34], surface antigen one (*sao*) [42], hyaluronidase (*hylA*) [17,43], opacity factor (*ofs*) [37], fibronectin binding protein (*fbps*) [44], arginin deiminase (*arcA*) [45], glyceraldehyde-3-phosphate dehydrogenase (*gapdh*) [46], regulator of virulence (*revS*) [35,47], enolase (*eno*) [48], glutamine synthetase (*glnA*) [49], *igA1 protease *[36], inosine 5-monophosphate dehydrogenase (*impdh*) [50], dipeptidyl peptidase IV (*dppIV*) [51], ferrous iron transporter (*feoB*) [52], subtilisin like serine protease (*sspA*) [53], amylopullulanase (*apuA*) [54], ferric uptake regulator (*fur*), and adhesion competence repressor (*adcR*) [55]. * *hylA *is present as pseudogene in P1/7 and does not have a SSU-number. '+' indicates all probes have a ratio > -1.5 (present); light grey shading indicates one or more probes have a ratio between -1.5 and -3 (present with slight variation); dark grey shading indicates one or more probes have a ratio between -3 and 4.5 (present with large variation); '-' indicates one or more probes have a ratio < -4.5 (partly or completely absent).

To correlate virulence of isolates with specific genes, we next studied the distribution of 25 genes encoding putative virulence proteins in serotype 2 isolates among isolates. Genes were selected that were described to be involved in pathogenesis or virulence of *S. suis*. Clustering of these results into a dendrogram assigned all isolates to 7 different virulence clusters (V1 - V7) (Figure 2). This clustering was very similar to the clustering based on the CGH data, although some isolates were clustered with isolates that belonged to another CGH cluster. Isolates assigned to cluster V4 (corresponding to CGH cluster A) contained all selected putative virulence genes, whereas isolates assigned to clusters V1, V2, V3, V5, V6 and V7 (corresponding to CGH cluster B) lacked 1 to 12 of these genes. All cluster B isolates lacked either one or more sortase genes that are involved in assembly of pili [31]. Serotype 7 isolates all clustered to V1 together with MRP^-^EF^- ^serotype 2 isolates. All V1 isolates lacked regulator of virulence *revS*, *epf *and *srtB *and *srtC*, whereas they contained *srtE, srtF *and two isolates contained *srtD*, but with extensive sequence variation. Serotype 9 isolates fell apart in two different clusters, V6 and V7. Cluster V6 lacked *IgA protease*, *srtF*, and *epf*, and showed minor sequence variation in *apuA *and *fbps*. V7 isolates lacked at least 11 putative virulence genes, among which all sortase genes. This indicated that V7 isolates are incapable of pilus formation, and are thereby likely to be less virulent. Taken together, our data suggests that differences in virulence exist within the serotype 9 population. Extensive sequence variation in a limited number of putative virulence genes (*glnA*, *ofs*, *IgA protease*, *apuA*, *fbps*, *srtD*) was detected in isolates belonging to clusters V1, V2, V3, V5, V6 and V7, but not in V4 isolates (Figure 2). This suggests that V4 isolates are genetically more similar to each other and to P1/7, the array strain. V4 isolates exclusively express EF, none of the isolates in clusters V1, V2, V3, V5, V6 express EF (Table 1). In this study we show that most isolates are unable to express the protein since they lacked the *epf *gene encoding EF. Two V5 isolates have a silent *epf *gene. Presence of *mrp *and *sly *genes was less indicative for protein expression. Isolates 3995, 3988, OV209, 15009, and 5973 contained the suilysin gene, but did not express the protein under *in vitro *conditions (Table 1). Almost all isolates tested in this study contained the *mrp *gene, whereas less than half expressed the protein under *in vitro *conditions (Table 1 and Figure 2) [13].

### Regions of differences and core genome of *S. suis*

To further explore genetic diversity between *S. suis *isolates, regions of difference (RDs) were identified, which were defined as at least three consecutive ORFs that were absent from at least one strain. Thirty-nine RDs that varied in size from 461 bp to 27 kbp were identified. The largest RD (27 kbp) contained *cps *genes encoding serotype specific polysaccharide capsule of P1/7 (serotype 2) (Table 3). Other RDs contained ABC transporters, restriction modification systems, signal peptidases (*srtE, srtF*), several transporters, two-component systems and several other genes (Table 3).

**Table 3:** Regions of difference (RDs) identified in relation to P1/7.

RD^#^	Range in P1/7*	Size (bp)*	Present in n/55 strains (parts present in n/55)	%GC^$^	Predicted Function*
RD01	SSU0101 - SSU0111	7.537	23 (49)	34.1	Integrase, replication initiation factor, hypothetical proteins
RD02	SSU0178 - SSU0182	5.501	47	40.8	PTS IIB, transketolase
RD03	SSU0198 - SSU0209	14.234	37 (13)	33.7	PTS IIABC transporter, glucosamine-6-phosphate isomerase, pseudogene
RD04	SSU0300 - SSU0305	5.455	36 (17)	43.0	Dehydrogenase, flavin oxidoreductase, transcription regulator lipase
RD05	SSU0346 - SSU0350	7.680	29	38.8	merR, hypothetical proteins
RD06	SSU0413 - SSU0418	8.624	29 (14)	33.6	Hypothetical proteins
RD07	SSU0423 - SSU0428	8.383	30 (11)	39.3	Signal peptidase, *srtF*
RD08	SSU0449 - SSU0453	2.475	52	36.0	Signal peptidase, *srtE*
RD09	SSU0519 - SSU0556	27.705	30 (6)	35.6	*cps*-genes, transposases
RD10	SSU0592 - SSU0600	8.410	52	36.7	Hypothetical proteins, D-alanine transport
RD11	SSU0640 - SSU0642	5.514	42	42.5	Type III RM
RD12	SSU0651 - SSU0655	7.674	34 (5)	38.8	Type I RM
RD13	SSU0661 - SSU0670	10.283	50	40.1	PTS IIABC, formate acetyltransferase, fructose-6-phaphate aldolase, glycerol dehydrogenase
RD14	SSU0673 - SSU0679	8.872	45	42.1	Piryidine nucleotide-disulphide oxidoreductase, DNA-binding protein, glycerol kinase, alpha-glycreophophate oxidase, glycerol uptake facilitator, dioxygenase
RD15	SSU0684 - SSU0693	7.868	35	38.6	Phosphatase, phosphomethylpyrimidine kinase, hydroxyethylthiazole kinase, thiamine-phosphate pyrophosphorylase, uridine phosphorylase, cobalt transport protein, ABC transporter
RD16	SSU0804 - SSU0815	11.036	20	30.6	Plasmid replication protein, hypothetical proteins
RD17	SSU0833 - SSU0835	2.386	31	34.1	Lantibiotic immunity
RD18	SSU0850 - SSU0852	2.345	50	40.9	Pyridine nucleotide-disulphide oxidoreductase, hypothetical proteins
RD19	SSU0902 - SSU0904	2.169	52	36.4	Hypothetical proteins
RD20	SSU0963 - SSU0968	2.769	54	43.2	Acetyltransferase, transposases
RD21	SSU0998 - SSU1008	13.688	54	42.3	Glycosyl hydrolase, UDP-N-acetylglucosamine 1-carboxyvinyltransferase, 2-deoxy-D-gluconate 3-dehydrogenase, mannonate dehydratase, urinate isomerase, 2-dehydro-3-deoxy-6-phosphogalactonate aldolase, beta-glucuronidase, carbohydrate kinase, sugar transporter
RD22	SSU1047 - SSU1066	17.452	52	40.1	Hyaluronidase, PTS IIABCD, aldolase, kinase, sugar-phosphate isomerase, gluconate 5-dehydrogenase, transposase
RD23	SSU1169 - SSU1172	4.850	53 (1)	42.6	ABC transporter
RD24	SSU1271 - SSU1274	6.695	36 (1)	35.8	Type I RM
RD25	SSU1285 - SSU1287	805	43	41.7	Hypothetical proteins
RD26	SSU1308 - SSU1310	4.130	52	36.7	PTS IIABC
RD27	SSU1330 - SSU1347	10.041	28	37.1	Phage proteins, hypothetical proteins
RD28	SSU1369 - SSU1374	7.733	53	38.8	Sucrose phosphorylase, ABC transporter
RD29	SSU1402 - SSU1407	5.018	29 (24)	41.2	Bacitracin export, transposase
RD30	SSU1470 - SSU1476	10.163	52	35.4	Two-component regulatory system, serum opacity factor
RD31	SSU1588 - SSU1592	7.771	52	40.9	Type I RM, integrase
RD32	SSU1702 - SSU1715	23.640	45	43.4	Two-component regulatory system, tranpsoase, glucosaminidase, hypothetical proteins, alpha-1,2,-mannosidase, eno-beta-N-acetylglucusaminidase
RD33	SSU1722 - SSU1727	4.924	30	38.3	Acetyltransferase, hypothetical proteins, PTS IIBC
RD34	SSU1763 - SSU1768	6.153	29	47.1	Nicotinamide mononucleotide transporter, transcriptional regulator, hypothetical proteins
RD35	SSU1855 - SSU1862	8.479	52	39.9	PTS IIABC, hypothetical proteins, beta-glucosidase, 6-phospho-beta-glucosidase
RD36	SSU1872 - SSU1875	1.918	36	35.4	RevS, CAAX amino terminal protease
RD37	SSU1881 - SSU1890	13.184	36	38.5	srtB, C, D
RD38	SSU1927 - SSU1931	8.444	44	41.6	β-glucosidase, two-compent regulatory system
RD39	SSU1942 - SSU1944	461	42	40.5	mutT/NUDIX hydrolase

^# ^Region of difference is defined as regions of at least 3 ORFs that are absent from at least 1/55 *S. suis *strains tested.

* Naming, size and function prediction is based on genome sequence of P1/7 [7]

^$ ^GC-percentage of P1/7 genome is 41%

**Figure 3:**
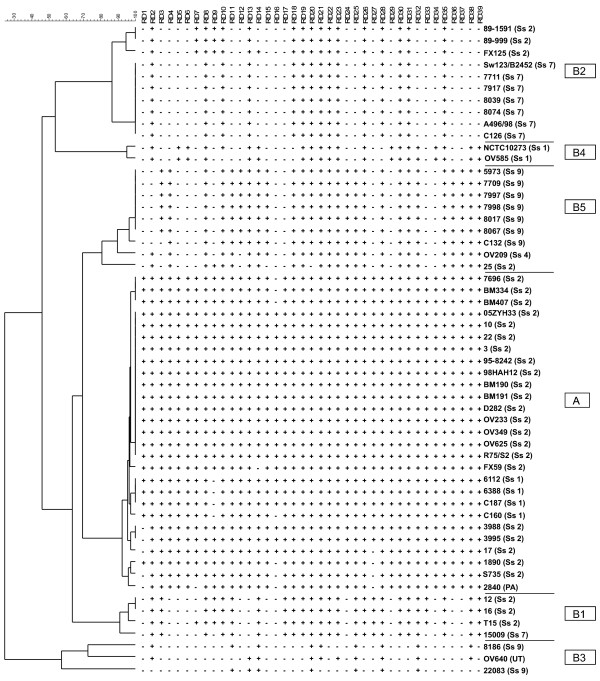
**Dendrogram based on the presence/absence of regions of difference (RD) among *S. suis *isolates**. RDs were defined as at least three consecutive ORFs that were absent from at least 1 strain. Naming of clusters is corresponding to the CGH clustering.

Clustering of RD distribution among isolates in a dendrogram resulted in an identical clustering compared to CGH clustering, indicating that RDs mainly determine the differences between isolates as detected by CGH (Figure 3). Within cluster A, subclusters could not be discriminated based on the absence/presence of specific RDs, since most RDs were universally present within cluster A isolates. Distribution of RDs among cluster B was more heterogeneous. Three isolates from cluster B3 (22083^R1^, 8186 and OV640) were responsible for a good deal of diversity: 9 RDs representing 45 genes were only absent in one or more of these isolates; whereas in total at least 29 RDs are missing from these isolates. Thus, these isolates are atypical within our selection of isolates. Serotype 7 and 9 isolates (in clusters B2 and B5) also lacked considerable numbers of RDs. For some RDs (RD1, RD6, RD17), GC content differed considerably from overall GC content of the genome (41%), indicating these RDs might have been acquired from other species by horizontal gene transfer, since foreign DNA can often be recognized by its variation from the majority of the genome in base composition or codon preference. The gene content of RDs shows that these regions contain specific beneficial traits like RM systems, ABC transporters, or two-component systems, making it attractive regions to acquire.

**Figure 4:**
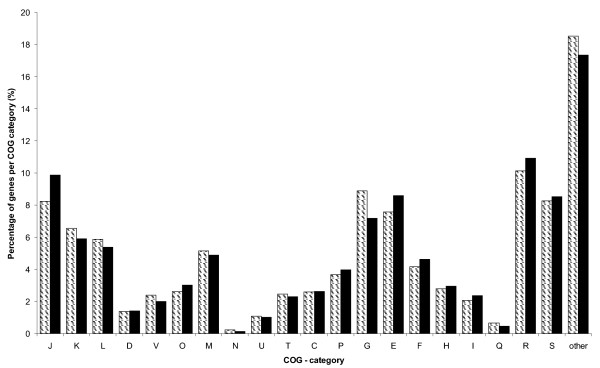
**Representation of COG categories among the core genome**. Relative representation of COG categories in the whole genome (hatched bars) compared to the core genome (black bars) of *S. suis *strain P1/7. Representation is calculated as the percentage of genes per COG category compared to the total number of genes in the genome. COG categories: J translation, ribosomal structure and biogenesis; K transcription; L replication, recombination and repair; D cell cycle control, cell division, chromosome partitioning; V defense mechanisms; O posttranslational modification, protein turnover, chaperones; M cell wall/membrane/envelope biogenesis; N cell motility; U intracellular trafficking, secretion, and vesicular transport; T signal transduction mechanisms; C energy production and conversion; P inorganic ion transport and metabolism; G carbohydrate transport and metabolism; E amino acid transport and metabolism; F nucleotide transport and metabolism; H coenzyme transport and metabolism; I lipid transport and metabolism; Q secondary metabolites biosynthesis, transport and catabolism; R general function prediction only; S function unknown; 'other' no COG category attached.

A core genome for *S. suis *was defined by selecting genes that were present in all *S. suis *isolates tested. The resulting core genome of *S. suis *consisted of 1492 genes (76%) out of 1960 genes present on our array. Of those 1492 genes, 26 genes represent pseudogenes in P1/7. Composition of the core genome of *S. suis *was studied using the classification in clusters of orthologous groups of proteins (COG). Figure 4 displays the relative representation of each COG category in both P1/7 as well as in the core genome. Most COG categories were equally represented in both genomes. However, COG categories J (translation, ribosomal structure and biogenesis), E (amino acid transport and metabolism) and F (nucleotide transport and metabolism) were found to be overrepresented in the core genome. In conclusion, all isolates in our study share 1492 genes. The overrepresentation of the structural gene categories J, E, and F suggest this core genome suffices for growth, division and survival, whereas additional, beneficial traits are mainly encoded by RDs.

## Discussion

Comparative genome hybridization (CGH) was used to study genetic heterogeneity among a collection of 55 *S. suis *isolates. *S. suis *isolates were assigned to two clusters (A and B).

CGH data was compared with MLST and pulse field gel electrophoresis (PFGE) [6] and amplified fragment length polymorphism (AFLP)[25]. In general there was a lot of congruence between typing methods. The discriminatory power of CGH is larger than that of MLST analysis, since isolates that belong to MLST CC1 can be divided into subclusters using CGH. Moreover, Vietnamese isolates that belong to different pulse field types, were assigned to the same CGH subcluster [6]. This could be explained by genomic inversions and substitutions, that were observed in the genome of the Vietnamese reference strain BM407 in comparison to P1/7 [7]. These changes can be discriminated by PFGE, but not by CGH.

To correlate virulence of isolates to CGH results, virulence of serotype 1 and serotype 9 isolates was determined in an experimental infection. For serotype 1, our animal experiment showed that in contrast to the field isolates, the reference strain was not highly virulent. Since serotype 9 only induced clinical symptoms at very high doses, we concluded that serotype 9 isolates were avirulent under experimental conditions. This was confirmed by other studies [32,33]. To correlate virulence to CGH data, distribution of 25 putative virulence genes among *S. suis *isolates was studied. Each CGH cluster was shown to be associated with a specific profile of putative virulence genes. Cluster A isolates contained all 25 putative virulence genes. Cluster B isolates on the contrary lacked up to 12 putative virulence genes among which one or more of sortase genes (*srtBCD*, *srtE*, *srtF*) that are involved in assembly of pili [31,34]. In agreement with data presented here, Takamatsu *et al*. showed that CC1 isolates contained all *srt *genes, whereas CC29 isolates lacked *srtBCD *genes [34]. However, none of our serotype 9 isolates contained the *srtBCD *gene cluster, whereas this cluster was detected in a Japanese serotype 9 isolate [34]. This could imply geographical variation. Moreover, the *revs *gene is absent from all cluster B isolates, with the exception of cluster B5 isolates. This regulator influences expression of putative virulence factors [35]. Therefore, lack of *revs *might affect virulence of isolates. The *IgA1 protease *gene was found to be absent in all serotype 9 isolates, and displayed extensive sequence variation in serotype 7 isolates. All serotype 2 isolates including the avirulent isolates contained the *IgA1 protease *gene. Zhang *et al*. showed that most pathogenic serotype 2 isolates contained the *IgA1 protease *gene, whereas the gene was sparsely found in non-invasive serotype 2 isolates [36]. In the latter study mainly isolates obtained in China were used. Sequence variation among isolates belonging to cluster B was observed for other putative virulence genes as well, like *ofs*, *glnA*, *fbps *and *apuA*. The *ofs *gene was highly conserved among virulent serotype 1 and 2 isolates but showed extensive sequence diversity in avirulent serotype 2 and serotype 7 isolates, as was also described by Takamatsu *et al *[15]. Interestingly, at least two of the *ofs *positive serotype 7 strains do not express OFS *in vitro*, as shown in the serum opacification assay [37]. This suggests the presence of silent *ofs *genes. A silent *epf *gene was present in isolates in cluster B3. Two of the B3 isolates (22083^R1 ^and 8186) expressed the enlarged version of MRP, but none of the probes used for the CGH hybridized to the *mrp *gene, suggesting extensive sequence variation exists between different serotype 9 isolates. The presence of a *mrp *gene in the two isolates was confirmed by PCR analysis (data not shown). Serotype 9 isolates were distributed among 2 virulence clusters, V6 and V7 that differed considerably in their distribution of putative virulence genes. This suggests differences in virulence exist among serotype 9 isolates that were not identified in our experimental infection model.

Avirulent MRP^-^EF^- ^serotype 2 isolates clustered together with serotype 7 isolates both by CGH as well as by MLST. Such a clustering is in agreement with previous studies [24,25]. The clustering strongly suggests similarity in genetic background between the isolates and could suggest that the avirulent serotype 2 isolates originated from serotype 7 isolates after the exchange of the capsular genes. Capsular exchange has been described for other streptococci like GBS [38] and *Streptococcus pneumonia *[39]. In this study 39 regions of differences (RDs) were identified, that might contribute to virulence or survival in the host based on the predicted functions of the genes associated with the various RDs. For example 3 RDs encode two-component regulatory systems and 5 RDs encode putative virulence genes. In addition, 6 phosphotransferase systems, and 4 ABC transporters were identified. Since the GC content of some RDs differed considerably compared to the whole genome of *S. suis*, these RDs could have originated from horizontal gene transfer. This suggestion can be supported by the finding that many RDs contained transposases, integrases or phage proteins which are all involved in gene transfer.

A core genome for *S. suis *was defined that contained 78% of P1/7 ORFs. This percentage is in the same order of magnitude as for other streptococcal core genomes. A small percentage (2.4%) of the core genome is represented by pseudogenes in P1/7. Since single nucleotide differences cannot be detected using CGH, additional putative pseudogenes present in other isolates will not be identified. This could lead to a small overestimation of the core genome. In P1/7 COG category G, carbohydrate transport and metabolism, is overrepresented compared to the core genome. This could reflect genes that are not essential to *S. suis*, but make *S. suis *strains carrying these gene(s) more versatile in their carbon source usage. Recent publications suggest carbon source usage may be an important virulence trait for streptococci [40], which implies the more versatile *S. suis *isolates could benefit in pathogenesis. Since the core genome includes genes that are shared by all isolates included in our study, representing virulent as well as avirulent isolates, it is not very likely the core genome alone is sufficient for virulence. This is confirmed by the finding of several genes putatively involved in virulence in the RD regions of P1/7 that probably attribute to virulence or survival in the host of P1/7. However, since all isolates, including avirulent ones like T15, 12 and 16 [13,21], can colonize porcine tonsils, the core genome might be sufficient for colonization.

## Conclusions

In conclusion, we show that CGH is a valuable method. Not only can it be used for genotyping of *S. suis *isolates, but CGH also gives information on phylogeny of isolates, and can be used to look for specific gene content, like virulence genes, or sequence variation among isolates. At present a disadvantage of CGH using the current microarray is the one way character of the technology; only distribution of genes present in P1/7 can be studied using the current microarray. Recently, several *S. suis *isolates have been sequenced adding new information to the *S. suis *pangenome. The Chinese human isolates were shown to contain an additional putative pathogenicity island (PI) of 89 kb compared to P1/7 [41], whereas the Vietnamese strain BM407 contained another additional PI compared to P1/7 [7]. Both PI's were shown to contain integrative and conjugative elements (ICE) not present in P1/7. The current microarray will have to be extended with genome sequences of other *S. suis *isolates to be a better representation of the *S. suis *pangenome.

## Conflicts of interests

The authors declare that they have no competing interests.

## Authors' contributions

AG carried out the molecular experiments, data analyses and drafted the manuscript. HJW collected the *S. suis *isolates and participated in the experimental infection. FMB performed statistical analysis of clustering methods. CS collected the Vietnamese isolates and helped to draft the manuscript. CGB collected and analyzed German isolates and helped to draft the manuscript. HNT analyzed the Vietnamese isolates. NSZ performed the experimental infections. HES initiated and coordinated the work described and helped to draft the manuscript. All authors read and approved the final manuscript.
